# Effect of a GLP-1 mimetic on the insulin response to oral sugar testing in horses

**DOI:** 10.1186/s12917-022-03394-2

**Published:** 2022-07-29

**Authors:** Darko Stefanovski, Mary A. Robinson, Andrew Van Eps

**Affiliations:** 1 Department of Clinical Studies, School of Veterinary Medicine, University of Pennsylvania, New Bolton Center Campus, 382 West Street Road, Kennett Square, PA 19348 USA; 2 PA Equine Toxicology & Research Laboratory, 220 East Rosedale Avenue, West Chester, PA 19382 USA

**Keywords:** Horse, Insulin dysregulation, Insulin sensitivity, Equine Metabolic Syndrome (EMS), Insulin secretion

## Abstract

**Background:**

Insulin dysregulation (ID) is the most important risk factor for the development of laminitis in horses and therapies to control it are needed.

**Hypothesis/objectives:**

To assess the effects of a single dose of the synthetic GLP-1 analog exenatide on postprandial insulin dynamics. We hypothesized that exenatide would improve insulin sensitivity and lower postprandial blood insulin concentrations.

**Study design:**

Randomized, crossover, experimental study.

**Animals:**

Six horses (3 mares, 3 geldings; 2 with normal insulin regulation [NIR] and 4 with mild ID).

**Methods:**

Horses completed both study arms: subcutaneous administration of exenatide (or no treatment) 30 min before an oral sugar test (0.15 ml/kg of Karo Syrup). Blood samples obtained over 240 min were assayed for glucose, insulin, lactate, c-peptide and total GLP-1. The area under the curve (AUC) was calculated using the trapezoidal rule. Insulin sensitivity (*S*_*I*_) was estimated using a mathematical model.

**Results:**

Exenatide resulted in a postprandial decrease of 20% (effect size: 2673 µU·min/ml; 95% CI: 900 – 4446 µU·min/ml; *P* = 0.003) in AUC of plasma insulin (control; mean AUC insulin: 11,989 µU·min/ml; 95% CI: 9673 – 14,305 µU·min/ml, exenatide; mean AUC insulin: 9316 µU·min/ml; 95% CI: 7430 – 11,202 µU·min/ml). Exenatide resulted in an approximately threefold increase (effect size: 5.56 10^–4^· µU/ml^−1^·min^−1^; 95% CI: 0.95 – 10.1 10^–4^· µU/ml^−1^·min^−1^; *P* = 0.02) in estimated insulin sensitivity (control mean *S*_*I*_: 1.93 10^–4^· µU/ml^−1^·min^−1^; 95% CI: 0.005 – 3.86 10^–4^·µU/ml^−1^·min^−1^ vs. exenatide mean *S*_*I*_: 7.49 10^–4^· µU/ml^−1^·min^−1^; 95% CI: 3.46 – 11.52 10^–4^· µU/ml^−1^·min^−1^).

**Conclusions:**

The decrease in insulin response to carbohydrates was due to an increase in whole-body insulin sensitivity. GLP-1 agonists may have therapeutic potential for ID in horses.

## Introduction

Insulin dysregulation (ID) is associated with increased risk of laminitis in horses [1] and experimental evidence suggests that hyperinsulinemia directly leads to lamellar damage [2, 3] possibly via activating insulin-like growth factor (IGF-1 [4]). In horses and ponies with ID, hyperinsulinemia is most pronounced in association with carbohydrate ingestion [5]; therefore, understanding and controlling the postprandial insulin response is critical to developing effective laminitis prevention and management strategies for horses with ID. Consumption of nutrients stimulates the secretion of gut originating hormones such as the glucagon-like peptide 1 (GLP-1) and glucose-dependent insulinotropic polypeptide (GIP) that have been termed incretin hormones for their ability to promote insulin secretion and thus potentially lead to hyperinsulinemia in humans [6]. A functional enteroinsular axis has been demonstrated in horses [7] and there is evidence suggesting that an increase in circulating GLP-1 may be partially responsible for differences in postprandial hyperinsulinemia observed between different breeds [8].

GLP-1 analogs have been at the forefront of therapy for Type 2 Diabetes in humans, due to their observed incretin effect: augmentation of glucose-dependent insulin secretion in the postprandial period [9]. GLP-1 analogs would be expected to exacerbate postprandial hyperinsulinemia in horses with ID, however incretin-independent actions have been demonstrated in other species for GLP-1 analogs such as exenatide [10]. Indeed, subcutaneous exenatide improved insulin sensitivity in normal dogs, independent of changes in insulin secretion [11]. Furthermore, in humans it has been show that GLP-1 analogs can slow gastric emptying, decrease the rate of glucose absorption and subsequently blunt the postprandial insulin response [12]. On the basis of these effects, the GLP-1 agonist semaglutide has been approved for the treatment of obesity in humans without type 2 diabetes [13].

A recent study demonstrated the presence of GLP-1 receptors in a wide range of equine tissues, suggesting that it is likely GLP-1 also has incretin-independent actions in the horse [14]. In addition, the same study showed that exenatide (in contrast to the effects of human GLP-1) did not stimulate secretion of insulin in equine isolated pancreatic islets [14]. It is therefore unclear what effects synthetic GLP-1 agonists may have when administered to horses, however their therapeutic potential warrants investigation. The objective of this study was to assess the effect of a single dose of exenatide (a GLP-1 analog) on insulin and glucose dynamics in horses after the administration of an oral sugar bolus. We hypothesized that in horses, exenatide would improve insulin sensitivity and reduce hyperinsulinemia.

## Results

No significant differences between the control and treatment arm of the experimental protocol were noted regarding the fasting baseline (0 min) values of glucose, lactate, insulin, and c-peptide.

In the control arm, 3 horses were classified as ID based on the plasma samples obtained in the fasting basal period. After the administration of OST, 4 horses were classified as ID based on results of 60 and 90 min samples (Table 1). In the treatment arm and based on the fasting basal samples obtained before the start of the OST, the same 3 animals that were classified as ID in the control arm again were classified as ID. No animals were classified as ID based on the samples obtained during the treatment arm OST (Table 1).

**Table 1:** Plasma insulin levels for each individual horse before, 60 min and 90 min after initiation of the oral sugar test (OST) for treatment (exenatide) and control (no treatment) periods

**Control period OST**						
	**Plasma Insulin [µU/ml]**					
**Horse**	**0 min**		**60 min**		**90 min**	
1	**27.4**	^*^	**80.9**	^*^	**52.3**	^*^
2	**25.3**	^*^	**86.8**	^*^	**65.3**	^*^
3	12.9		29.3		27.1	
4	**22.8**	^*^	**78.9**	^*^	**75.9**	^*^
5	12.8		**97.6**	^*^	**88.5**	^*^
6	6.4		30.2		29.0	
**Treatment period OST**						
	**Plasma Insulin [µU/ml]**					
**Horse**	**0 min**		**60 min**		**90 min**	
1	**21.9**	^*^	42.7		38.5	
2	**27.9**	^*^	19.6		29.2	
3	9.0		23.4		14.5	
4	**28.7**	^*^	30.2		33.8	
5	16.3		41.5		25.9	
6	7.6		17.5		14.0	

*Plasma insulin levels indicating insulin dysregulation (ID)

**Fig. 1:**
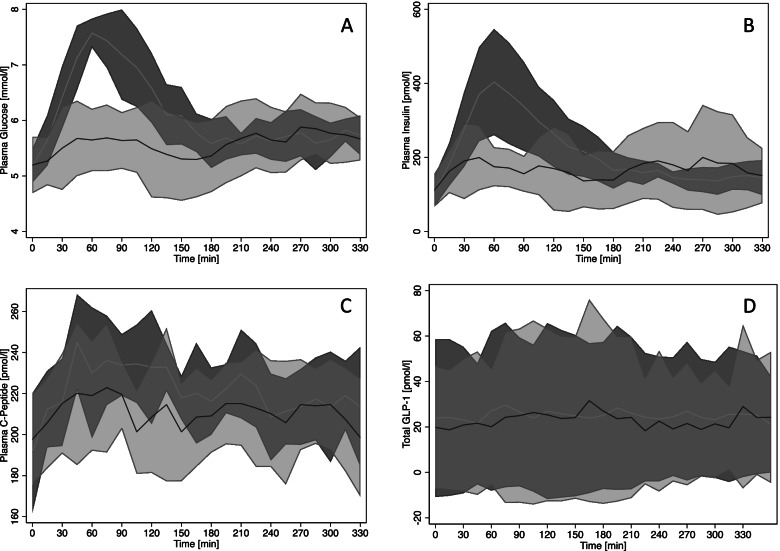
Temporal profile of plasma glucose (**A**), insulin (**B**) and c-peptide (**C**) and total GLP-1 (**D**) during the oral sugar test (OST). The dark shaded area (with mean represented with light grey line) indicates the 95% confidence interval of the average without treatment whilst the light shaded area indicates the 95% confidence interval for the average (dark grey line) with exenatide treatment

**Fig. 2:**
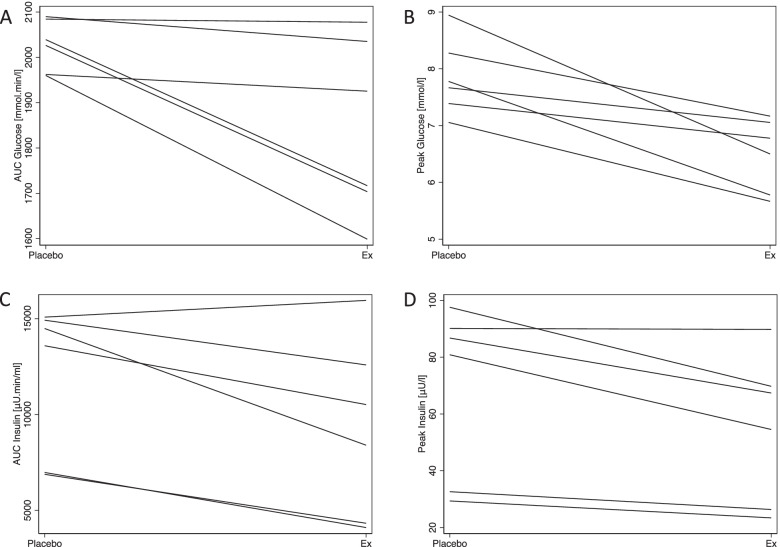
Spaghetti plots of changes with treatment per horse in area under the curve (AUC) of plasma glucose (**A**), peak plasma glucose (**B**), AUC plasma insulin (**C**), peak plasma insulin (**D**). The AUC of glucose was significantly decreased (*P* = 0.007, panel **A**) with treatment. Furthermore, the peak plasma glucose concentrations during the oral sugar test (OST) were also significantly reduced in the treatment group (*P* < 0.001; panel **B**). The AUC of insulin was significantly higher (*P* = 0.003; panel **C**) in the control period in comparison to the treatment. The peak insulin concentration (C_max_) during the OST was significantly reduced with treatment by 14.35 µU/ml (*P* = 0.003; panel **D**)

The model adjusted marginal mean calculated values for glucose, lactate, insulin, c-peptide and total GLP-1 are shown in Table 2. The area under the curve (AUC) of glucose was significantly decreased (effect size: 184 mmol·min/l; 95% CI: 50 – 318 mmol·min/l; *P* = 0.007; Figs. 1A; 2A; Table 2). Furthermore, the peak plasma glucose concentrations during the OST were also significantly reduced in the treatment group (effect size: 1.36 mmol/l; 95% CI: 0.77 – 1.96 mmol/l; *P* < 0.001; Figs. 1A; 2B; Table 2;). The AUC of lactate was not significantly altered with treatment (*P* = 0.3). The AUC of insulin was significantly higher (effect size: 2673 µU·min/ml; 95% CI: 900 – 4446 µU·min/ml, *P* = 0.003; Figs. 1B; 2C; Table 2) in the control period in comparison to the treatment. The peak insulin concentration (C_max_) during the OST was significantly reduced with treatment (effect size: 14.35 µU/ml; 95% CI: 5.01 – 23.69 µU/ml; *P* = 0.003; Figs. 1B; 2D; Table 2;). The AUC of c-peptide was not significantly different (*P* = 0.3; Fig. 1C; Table 2) between the two periods. The peak plasma c-peptide concentrations during the OST were also not significantly altered by treatment (*P* = 0.4; Fig. 1C; Table 2). AUC of GLP-1 showed a considerable level of variability resulting in wide 95% CI (control mean AUC Total GLP-1: 9046 pmol·min/l; 95% CI: 1563 – 16,530 pmol·min/l; exenatide mean AUC Total GLP-1: 7132 pmol·min/l; 95% CI: 485 – 13,779 pmol·min/l; Fig. 1D; Table 2). Nevertheless, The AUC of the treatment group was significantly reduced (effect size: 1914 pmol·min/l; 95% CI: 122 – 3706 pmol·min/l; *P* = 0.04) in comparison to the control group. Furthermore, the time to peak of total GLP-1 concentration was significantly (effect size: 145; 95% CI: 53.38 – 236.61 min; *P* = 0.002) altered where it took almost twice as long in the exenatide group in comparison to the control to reach its peak concentration.

**Table 2:** Marginal (model adjusted) mean comparison for calculated values of area under the curve (AUC), peak plasma concentration (Cmax), Time to Cmax for glucose, lactate, insulin, c-peptide and total GLP-1. Last row shows the marginal mean of insulin sensitivity estimate (*S*_*I*_) for the two arms of the experimental protocol

Parameter	Control			Exenatide			*P*
	**Marginal Mean**	**95% CI**		**Marginal Mean**	**95% CI**		
**AUC Glucose (mmol**·**min/l)**	2026	1948	2106	1843	1759	1927	**0.007**
**C**_**max**_** Glucose (mmol/l)**	7.85	7.26	8.44	6.49	6.14	6.84	** ≤ 0.001**
**AUC Lactate (mmol**·**min/l)**	157	140	175	143	121	166	0.3
**C**_**max**_** Lactate (mmol/l)**	0.70	0.64	0.76	0.62	0.54	0.69	0.2
**AUC Insulin (µU**·**min/ml)**	11,989	9673	14,305	9316	7430	11,202	**0.003**
**C**_**max**_** Insulin (µU/ml)**	69.55	49.86	89.24	55.17	39.41	70.99	**0.003**
**AUC c-peptide (pmol**·**min/l)**	73,382	68,876	77,888	69,831	63,931	75,730	0.3
**C**_**max**_** c-peptide (pmol/l)**	257.08	230.90	264.49	247.70	230.90	264.49	0.4
**AUC Total GLP-1 (pmol**·**min/l)**	9046	1563	16,530	7132	485	13,779	**0.04**
**Time to C**_**max**_** Total GLP-1 (min)**	188	135	240	333	290	375	**0.002**
***S***_***I***_*** (10***^***–4***^· ***µU/ml***^***−1***^·***min***^***−1***^***)***	1.93	0.01	3.86	7.49	3.46	11.52	**0.02**

**Fig. 3:**
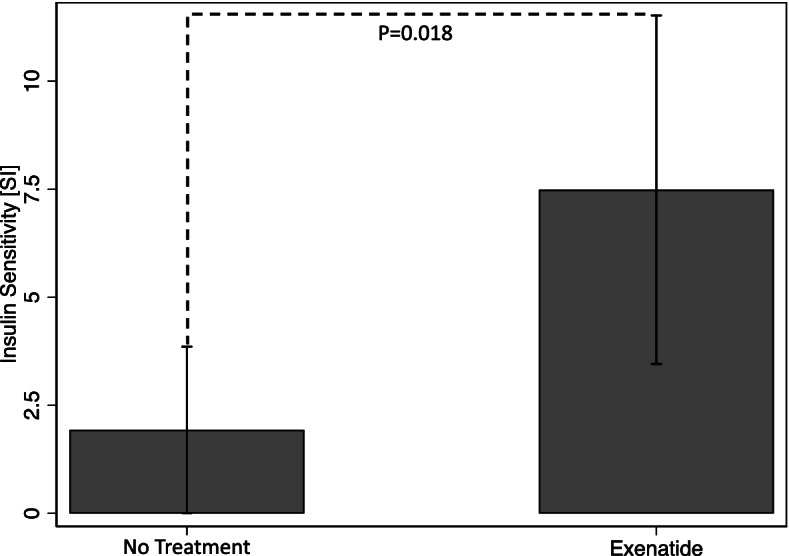
Bar graph of the marginal insulin sensitivity (*S*_*I*_) values for the placebo and exenatide treatment. Estimates of *S*_*I*_ were significantly higher (*P* = 0.02) after treatment with exenatide (mean *S*_*I*_: 7.49 10^–4^· µU/ml^−1^·min^−1^; 95% CI: 3.46 – 11.52 10^–4^· µU/ml^−1^·min^−1^) in comparison to control (mean *S*_*I*_: 1.93 10^–4^· µU/ml^−1^·min^−1^; 95% CI: 0.005 – 3.86 10^–4^· µU/ml^−1^·min^−1^)

Estimates of *S*_*I*_ were significantly higher (effect size: 5.56 10^–4^· µU/ml^−1^·min^−1^; 95% CI: 0.95 – 10.1 10^–4^· µU/ml^−1^·min^−1^; *P* = 0.02; Fig. 3; Table 2) after treatment with exenatide (mean *S*_*I*_: 7.49 10^–4^· µU/ml^−1^·min^−1^; 95% CI: 3.46 – 11.52 10^–4^· µU/ml^−1^·min^−1^) in comparison to control (mean *S*_*I*_: 1.93 10^–4^· µU/ml^−1^·min^−1^; 95% CI: 0.005 – 3.86 10^–4^·µU/ml^−1^·min^−1^).

## Discussion

Our current data indicate that in a small group of horses (2 with NIR and 4 with mild ID), at odds with previous observations in other species [7] that exenatide should act as insulin secretagogue, single-dose administration of 0.02 ug/kg of the GLP-1 agonist exenatide, 30 min prior to an OST led to a significant reduction in the plasma insulin concentration and 20% lower C_max_ during the OST. For the four horses that were classified as ID in the control arm, the treatment resulted in normalization of their plasma insulin concentrations during the OST, below published cutoff values for classification as ID [15]. The high level of variability (and relatively poor repeatability) of the OST [16, 17] further emphasizes the strength of these findings, as the effect identified was sufficiently large relative to the variance of the OST to reach significance. Furthermore, the treatment with exenatide also resulted in a 9% reduction in the AUC postprandial glucose concentration and a 17% lower peak glucose level. There are two previously observed mechanisms of acute action of GLP-1 and its analogs that may explain our results.

First, it has been hypothesized that GLP-1, besides exhibiting acute incretin function, also augments the peripheral tissue uptake of insulin [18] and the peripheral insulin sensitivity [19]. Insulin exhibits a strong vasodilation function, which facilitates the delivery of oxygen, nutrients, and insulin to myocytes and is considered another mechanism of enhanced insulin sensitivity [20]. It has been shown that GLP-1 augments this function [21]. Furthermore, GLP-1 agonists can augment insulin sensitivity at the cellular level by increasing the insulin-stimulated cell-surface GLUT4 concentration [22]. In humans and dogs, the liver itself extracts a significant portion (more than 50%) of the insulin in the portal vein and a carbohydrate-rich meal increases this proportion [23]. Also, insulin in the liver directly suppresses endogenous glucose production [24]. It has been shown that a GLP-1 agonist results in an increase in the concentration of insulin extracted by the liver [25] and as such is contributing toward enhanced hepatic insulin sensitivity. The observed reduction in glucose and insulin in the current study both suggest that exenatide augmented the uptake of insulin and nutrients in the periphery (muscle tissue), as is the case in humans and murine models and as such had enhanced whole-body insulin sensitivity. This is also consistent with the findings of this study that exenatide treatment in horses resulted in increased insulin sensitivity.

Second, GLP-1 has been shown to have an impact on acute gastric motility in other species. In obese humans, 30 days of treatment with exenatide resulted in a significant gastric slowdown, which in turn is associated with loss of weight and improved glucose homeostasis [26]. Thus, a slowdown in gut motility in horses administered exenatide may have resulted in slower glucose absorption and produced a more tempered insulin response as observed by the significant decrease in postprandial AUC of insulin and also a decrease in the C_max_ values. Previously in humans, plasma c-peptide concentrations and deconvolution techniques have been used to reconstruct the profile of insulin secretion, capitalizing on the fact that insulin and c-peptide are secreted in equimolar fashion and the notion that the kinetics of c-peptide are simpler and invariable across individuals [27]. Furthermore, due to differences in the kinetics of insulin and c-peptide, in humans the plasma concentrations of c-peptide are usually higher than the levels of insulin. However, our current data shows slightly altered relationship between c-peptide and insulin (Fig. 1C vs. B). In the horse, it appears that this ratio is reversed. Due to this observation and lack of data on the kinetics of c-peptide in horses, we decided to use a simpler approach evaluating the change in the total c-peptide concentration as an indicator of the total insulin secretion. We found no significant changes in terms of insulin secretion as indicated by the AUC of c-peptide, suggesting that the AUC of c-peptide after treatment with exenatide was unchanged. Thus, a major effect of exenatide on insulin secretion was unlikely (since insulin and c-peptide are secreted in equimolar amounts). This result leads us to speculate that perhaps altered glucose absorption, as a result of slowed gastric emptying, may be more plausible in the horse.

Previously, Bamford and colleagues suggested that chronic increases in the release of GLP-1 in ponies and Andalusian horses may lead to increased insulin secretion and insulin resistance [8]. Thus, they suggested that perhaps increased insulin secretion brought forth by increased GLP-1 availability is responsible for the onset of insulin resistance in some horse. Our current data indicate that an acute, single dose of a synthetic GLP-1 agonist in a small group of Thoroughbred and Standardbred horses does not appear to increase insulin secretion. In our study, treatment with exenatide resulted in a significant twofold increase in insulin sensitivity in the treatment period that resulted in a significant decrease in postprandial AUC of insulin. Previously it has been shown in humans that exenatide does not alter secretion of GLP-1 [28]; however in the current study there was a small but significant observed decrease in total GLP-1 with the administration of exenatide (compared to control) in horses (Fig. 1D). Although not previously reported, it is possible that treatment with Exenatide may lead to acute decreased secretion of GLP-1 via a negative feedback loop and this could be partially responsible for lower insulin concentrations presumably due to decreased insulin secretion, however an effect on c-peptide concentrations would also have been expected and was not observed. It is also possible that the reduced plasma concentrations of endogenous GLP-1 occurred as a result of peripheral effects of exenatide such as slowed gastric emptying. Also, given the significant decrease in AUC of plasma insulin and the observed threefold increase in insulin sensitivity, it is likely that (at least in the short term) an exogenously imposed increase in GLP-1 activity does not result in obvious adverse metabolic effects (particularly exacerbation of hyperinsulinemia) at least in mixed cohort of normal horses and horses with mild ID. Interestingly, exendin-4 (the naturally occurring form of exenetide) did not stimulate equine pancreatic islets to secrete insulin in a recent in vitro study [14], whereas human GLP-1 did stimulate insulin release.

Our current study has several limitations. The experiments were conducted in a small cohort of horses, including some with ID. Although 4 of the 6 horses were “technically” classified as ID according to current guidelines [15, 29], the ID in these horses was very mild and perhaps not clinically relevant. Nevertheless, due to concern for possible exacerbation of hyperinsulinemia with exenatide administration (since it is generally used as an insulin secretagogue) horses with severe ID were not specifically recruited for this study. However, this study showed that regardless of the ID status of these animals, exenatide did not cause any increase in plasma insulin concentration at any point in time during the OST. Furthermore, treatment with exenatide reduced the insulin response to within normal limits in horses that were classified as ID based on the placebo arm of the current study. Also, only a single dose was administered and it is unclear what the effects of chronic dosing might be on insulin dynamics. We did not assess the PK/PD for exenatide since the plasma levels were not measured. Further studies evaluating the effects of exenatide on postprandial hyperinsulinemia (preferably after an actual meal, rather than the OST) and PK/PD of exenatide in horses with severe ID over extended periods are warranted. The current study did not include any evaluation of possible extra-pancreatic effects of exenatide, such as delayed gastric emptying. Future studies of exenatide in horses that incorporate a measurement of gastric emptying (such as a combined acetaminophen and meal challenge protocol [30] as recently described) would help to characterize the effects of exenatide in the horse. Total GLP-1 and not active GLP-1 was measured in the current study, therefore although there was an apparent decrease in endogenous GLP-1 concentration in the plasma, it is unclear whether there was an effect on circulating active GLP-1. However we were most concerned with whether exenatide affected the secretion of endogenous GLP-1 rather than its activity and total GLP-1 concentrations more closely reflect intestinal L-cell secretion of GLP-1 [31]. Ideally a placebo treatment (rather than no treatment) would have been used for comparison to exenatide, however the nature of the delivery device (multi-dose injector pen with 30 gauge needle) was difficult to replicate for placebo delivery. The act of administration itself using this device was very well tolerated and it is unlikely that stress from the procedure impacted the results since on both occasions (in the no administration and administration of exenatide arm) the needle was inserted in the subcutaneous tissue, however this cannot be completely discounted. We cannot determine from our data the duration of the effect of a single injection of exenatide on insulin dynamics. In this study, we administered exenatide 30 min before the OST. However, it is unclear if comparable effects will be achieved or if the exenatide will be efficacious if given with longer delay between dosing and a carbohydrate challenge. The total dose of exenatide administered in the current study was based on the highest dose prescribed for human use (10 mcg per dose, [32]), which is a conservative dose rate for the horse. Given the absence of adverse effects in this study, evaluation of the effect of higher dose rates may be warranted. In humans exenatide is recommended to be administered within 60 min prior to a meal [32] and in a canine study it has been administered 15 min prior to a mixed meal [33]. To allow time for distribution we decided to administer exenatide 30 min prior to the OST in the currently study, however the influence of the timing of administration on the insulin response to carbohydrate challenge remains unclear.

Ideally the frequently sampled insulin‐modified intravenous glucose tolerance test (FSIGTT) with minimal model analysis is used to quantify insulin sensitivity [34], however in the current study we opted instead to estimate it based on minimal model analysis of the OST. Since the FSIGTT essentially bypasses the enteroinsular axis, it was not considered to be as appropriate for this study, particularly considering that exenatide might potentially alter the absorption of glucose as it does in other species [18]. The use of minimal model analysis of OST results greatly reduced the expense and complexity of the experiments in the current study, compared to including multiple FSIGTT protocols. The model used in the current study has been validated in humans with both mixed-meal (various nutrient compositions) and oral glucose tolerance tests (OGTT [35],). Furthermore, it has been shown that there is high correlation between the OGTT and FSIGT test estimates of insulin sensitivity in humans [36]. This also warrants investigation specifically in the horse.

Regardless of the mechanism of action, our current results demonstrate that postprandial hyperinsulinemia may be reduced using a synthetic GLP-1 agonist and this may be useful for the therapy of insulin dysregulation in horses with ID. No ill effects from a single dose of exenatide were noted and this treatment may potentially be useful for reducing hyperinsulinemia in response to ingested carbohydrate in horses. Further research evaluating the safety and efficacy of GLP-1 analogs specifically in insulin dysregulated horses, is warranted.

## Methods

### Study design and animals

Six horses were randomly selected from our experimental heard at the Department of Clinical Studies – New Bolton Center, School of Veterinary Medicine, University of Pennsylvania, Kennett Square PA (Table 3). After the study was completed, it was established that 2 horse had NIR and 4 were with mild ID (Table 1). Each of the animals completed a randomized, crossover study, which consisted of the subcutaneous administration of a bolus of 0.02 µg/kg (0.04 ml) of exenatide (Byetta, AstraZeneca, Cambridge UK) or no treatment (control arm; subcutaneous penetration with a 30 gauge needle) 30 min before the oral administration of 0.15 ml/kg of Karo Syrup that initiates the OST, ACH Food Companies, Inc., Oakbrook Terrace IL. Based on previously published mean values of SI in horses [34] and assumed change in SI of 30%, power = 0.8, alpha = 0.05 and using power analysis for a two-sample paired-means test, we estimated the sample size to be *N* = 6 [34]. All procedures were approved by the Institutional Animal Care and Use Committee.

**Table 3:** Signalment for normal horses with varying degrees of insulin dysregulation (ID, *N* = 6)

**Signalment**	
N	6
Breed	
Standardbred	4
Thoroughbred	2
Sex	
Mare	3
Gelding	3
Age (years)	8.67 (1.43)
Body Weight (kg)	593 (19)

Horses were fasted for 12 h prior to the OST. Using a random number generator, each animal was initially assigned to no treatment or exenatide administration (*n* = 3 per treatment group in first arm of the protocol). Thirty minutes after the administration of exenatide (or no and no treatment group) the first OST was conducted. Following the initial OST, a 7-day washout period was performed prior to the second OST which also was initiated 30 min after the administration of exenatide. The assignment to one of the two groups (no treatment or exenatide) was reversed from the assignment during the initial, first OST. For blood sampling, each horse was aseptically instrumented with a 14-gauge intravenous catheter (Angiocath, Becton, Dickinson and Company, Franklin Lakes, NJ) in a jugular vein on the same day and prior to the OST and, blood samples were taken every 15 min in the interval between 0 (fasting basal sample prior to OST) and 330 min and stored into EDTA and sodium fluoride oxalate tubes. After collection, the tubes were centrifuged, and the plasma was transferred to a new set of 10 ml plastic tubes and stored in -80 C freezer.

Plasma samples were assayed for insulin using radioimmunoassay (RIA; Millipore Sigma, Merck KGaA, Darmstadt, Germany) at Cornell University’s endocrinology laboratory as previously described [37]. Glucose and lactate were measured using a Radiometer ABS800flex (Radiometer Medical ApS, Bronsoj, Denmark) and assay based on amperometric measuring principles and proprietary membrane chemistry. Plasma c-peptide was measured using human double-antibody RIA by the Diabetes Research Core at the University of Pennsylvania, Institute for Diabetes, Obesity and Metabolism (IDOM) as previously described [38]. Plasma total GLP-1 was measured using an ELISA (EZGLP1T-36 K, Millipore, Sigma) as previously described [7, 39]. The guidelines suggested by Restifo and colleagues were used to classify animals as ID based on plasma insulin concentration at 0 min (fasted state, insulin ≥ 20 μIU/mL) and 60 min or 90 min (fed state, insulin ≥ 45 μIU/mL) after the initiation of the OST [15, 29].

## Mathematical modeling

### Minimal model of glucose metabolism

All mathematical modeling was performed using WinSAAM mathematical modeling software (University of Pennsylvania, Kennett Square, PA). We used the Dalla Man’s et al*.* version of the OGTT minimal model, which was adapted for use in the horse [40]. Based on the experimental data for all animals, an average time profile for all animals was generated. The mathematical model was fitted to the averaged time profile using the human initial values for the adjustable parameters of the model. This process yielded initial estimates of the parameters which were used in estimating the relevant indices for each of the animals. The mathematical formulation of the OGTT model couples the minimal model of glucose kinetics developed for interpretation of FSIGTT data [41], with a novel model for describing glucose absorption, *Ra*. The oral minimal model has the following formulation,

1 $$\left\{\begin{array}{lc}\dot{G(t)}=\left(p_1+X\left(t\right)\right)G\left(t\right)+p_1G_b+\frac{Ra(t)}V;G\left(0\right)=G_b&\lbrack\frac{mg}{dl}/min\rbrack\\\dot{X(t)}=-p_2X\left(t\right)+p_3(I\left(t\right)-I_b;X\left(0\right)=0&\lbrack\frac1{{min}^2}\rbrack\end{array}\right.$$

Where $\dot{G}\left(t\right)$ is the glucose concentration at the time, *t*. The experiment is initiated with a glucose load where glucose at *t* = 0, G(0), is equal to the basal glucose. This is unlike the FSIGTT where it has to be assumed that there is instantaneous mixing of the glucose bolus into the circulation and that the experiment starts from a value *G0* different from the fasting value of glucose and slightly higher than the peak value of plasma glucose [41]. Furthermore, there was no need to exclude any of the observations in the initial 10 min (common practice when using the minimal model) as the instantaneous mixing assumption did not apply. Parameter *p*_*1*_ of the model is glucose effectiveness also abbreviated as parameter Sg. Previously, it has been shown [42] that glucose effectiveness is a fairly constant value across multiple populations. Hence, and to lower the number of parameters to be estimated, *p*_*1*_ has been fixed to a value of 0.014 1/min [40]. One shortcoming of our current study is that we adapted this assumption as we were not able to estimate this value in horse. Thus, we assume that that this value is similar in the horse and just as in humans, there is no major difference in the value among animals or animal cohorts. *X(t)*, represents insulin action and relates to the concentration of insulin in the periphery (remote compartment). Parameter *p*_*3*_ reflects the rate of appearance of insulin in the remote compartment. Parameter *p*_*2*_ represents the fractional rate of irreversible loss of insulin action from the remote compartment. Equation 1 also indicates that at basal insulin (*I*_*b*_), insulin action equals 0 (*X(0)* = *0*). Insulin sensitivity is calculated as the ratio of *p*_*3*_ and *p*_*2*_ (Eq. 2).

2 $${S}_{I}=\frac{{p}_{3}}{{p}_{2}}{[(\mathrm{mU}/\mathrm{l})}^{-1}{\mathrm{min}}^{-1}]$$

The mathematical model outlined in Eq. 1 is essentially Bergman’s minimal model ^1^. The novelty of Dalla Man’s et. al. approach is the mathematical formulation of the post-absorptive rate of glucose appearance in plasma (*Ra*).

To transform the units *Ra(t)* (Eq. 3), the rate of appearance of glucose in plasma is divided by the fixed bodyweight volume of distribution assumed to be *Vd* = 1.7 dl/kg. Furthermore, it is assumed that 86% of the initial carbohydrate load is absorbed by 420 min [40]. Both of these values (*Vd* and maximal carbohydrate load absorbed) were adopted from human literature and future studies will have to be designed with the purpose of estimating the bodyweight normalized glucose volume of distribution and the fraction of the initial load absorbed specific for horse.

3 $$Ra\left(t\right)=\left\{\begin{array}{c}{k}_{i-1}+\frac{{k}_{i}-{k}_{i-1}}{{t}_{i}-{t}_{i-1}}\left(t-{t}_{i-1}\right);{t}_{i-1}\le t\le {t}_{i};i=1..4\\ {k}_{4}\mathrm{exp}\left(-\alpha t\right);t>{t}_{4}\end{array}\right.\left[\frac{mg}{kg}/min\right]$$

*Ra(t)* was formulated as a piecewise linear regression. For points beyond 120 min, *Ra(t)* was portrayed as single exponential with a slope α fixed to a value 0.017 1/min.

## Statistical analysis

All analyses were conducted using statistical software (Stata 16MP, StataCorp LLC, College Station TX), with two-sided tests of hypotheses and a *p*-value < 0.05 as the criterion for statistical significance. Tests of normal distribution (Shapiro–Wilk test of normality) were performed to determine the extent of skewness. For data with normal distribution, mean and standard deviation were reported. Significantly skewed data were reported as medians and interquartile ranges (IQR). Frequency counts and percentages were used for categorical variables (e.g., sex, signalment and others).

The area under the curve (AUC) above zero for the plasma concentration time curve of insulin, c-peptide and insulin was calculated using the trapezoidal rule. The peak insulin (C_max_) concentration was also assessed.

Multilevel mixed-effects linear regression was used to identify any potential significant effects of the treatment on the outcomes of interest adjusted for age, weight, gender, and breed as significant confounders that were previously shown to be associated with insulin resistance [43]. Random effects were set on the level of the individual animals. To permit for departure from normality of the residuals, a robust estimation of the variance was used. *Post-hoc* tests were used to estimate the marginal means and differences. All results are reported as marginal means and differences with 95% CI. Fisher's protected least-significant difference was used to guard against an increased probability of Type 1 error due to multiple significance tests.

## Data Availability

The data supporting the findings of this study are available from the authors upon reasonable request.
